# The home physical activity environment as a potential mediator of the association between parental physical activity and preschoolers’ physical fitness: a cross-sectional study in urban China

**DOI:** 10.3389/fpubh.2026.1880097

**Published:** 2026-10-28

**Authors:** Haiyun Li, Weilun Zhao, Xinyi Hu, Fang Wan, Pengpeng Dong, Weimin Zhang

**Affiliations:** 1 Xuzhou Kindergarten Teachers College, Xuzhou, Jiangsu, China; 2 Department of Public Culture, Nanchang Health Vocational and Technical College, Nanchang, Jiangxi, China; 3 Faculty of Education, Nanchang Vocational University, Nanchang, Jiangxi, China

**Keywords:** China, ecological model, home environment, mediation analysis, parental physical activity, physical fitness, preschoolers

## Abstract

**Introduction:**

Family-level associations with preschool children’s physical fitness remain incompletely understood.

**Objective:**

This study examined cross-sectional associations among parental physical activity, the home physical activity environment (HPAE), and preschool children’s fitness, including a potential indirect association through the HPAE.

**Methods:**

We recruited 568 parent–child dyads from 23 kindergartens in Nanchang, China, using multistage stratified random sampling. Parental activity was assessed using the International Physical Activity Questionnaire-Short Form, the HPAE using a purpose-developed 18-item scale, and children’s fitness using an adapted Chinese National Physical Fitness Measurement framework. Mediation analysis used 5,000 bootstrap resamples. The primary model controlled for parental education and family income. A sensitivity model additionally controlled for children’s outdoor activity, organized sport participation, and screen time.

**Results:**

In the primary model, parental activity was positively associated with children’s fitness (path c, *β* = 0.167) and the HPAE (path a, *β* = 0.349), which was also associated with fitness (path b, *β* = 0.234; all *p* < 0.001). The indirect association was statistically significant (a × b = 0.081, 95% CI [0.055, 0.109]), and the direct association remained significant (path c′, *β* = 0.085, *p* = 0.006). The 48.9% mediated proportion applies only to the primary model. After additional adjustment for children’s outdoor activity, organized sport participation, and screen time, the estimated indirect association was close to zero and was not statistically significant (a × b = −0.002, 95% CI [−0.013, 0.007]).

**Conclusion:**

The primary model showed a pattern of associations consistent with a potential pathway through the HPAE, but the indirect association was not robust to adjustment for child activity variables. These cross-sectional findings do not establish a mediation mechanism. Longitudinal and experimental studies are needed to clarify temporal ordering and evaluate potential intervention targets.

## Introduction

The global decline in children’s physical fitness has become a pressing public health concern (1, 2). According to the World Health Organization, over 80% of adolescents worldwide fail to meet the recommended 60 min of daily moderate-to-vigorous physical activity, and this pattern of insufficient movement often takes root in the preschool years (3). In China, analyses of five successive waves of the National Physical Fitness Surveillance (conducted in 2000, 2005, 2010, 2014, and 2020) have revealed troubling trends among preschool-aged children, including rapidly rising obesity rates and stagnating performance on standardized fitness tests such as the standing long jump and the 10-meter shuttle run (4, 5). Because the preschool period represents a critical window for establishing lifelong movement habits and for the rapid maturation of fundamental motor skills, understanding the determinants of physical fitness during this developmental stage carries substantial implications for population health (6, 7).

Among the many factors that shape young children’s health behaviors, parents occupy a uniquely influential position. Social cognitive theory posits that children acquire behavioral repertoires through observational learning, modeling the attitudes, values, and actions of significant adults in their immediate environment (8). In the context of physical activity, parents who regularly engage in exercise are thought to transmit pro-activity norms to their children through both direct modeling and the creation of supportive conditions for movement (9, 10). Empirical research has consistently confirmed a positive, albeit modest, association between parental physical activity and children’s own activity levels. A systematic review by Petersen et al. (11) synthesizing 39 studies concluded that the majority reported weak-to-moderate positive correlations between parent and child physical activity regardless of the child’s age or the gender composition of the parent–child dyad. Similarly, Matos et al. (12) found consistent associations between parental and child physical activity among 6- to 12-year-olds, with same-gender modeling effects appearing particularly salient. Among preschool children specifically, Xu et al. (13) demonstrated that parental physical activity levels, encouragement, and facilitation were each independently associated with young children’s activity and sedentary behaviors.

This parent–child resemblance in activity- and fitness-related outcomes, however, is unlikely to reflect a single pathway. Beyond direct behavioral modeling, prior work points to several additional and potentially overlapping mechanisms through which physically active parents may be associated with fitter children, including shared socioeconomic resources, parental health beliefs and literacy, general parenting practices and warmth, co-participation and shared family routines, active transportation and screen-time rules, provision of activity opportunities such as enrollment in sport programs, and heritable fitness-related traits (9, 14, 15). Genetic and shared-environment influences in particular mean that parent–child similarity in fitness cannot be assumed to arise solely from proximal behavioral transmission. Against this broader backdrop, the present study focuses specifically on one theoretically prominent and modifiable candidate pathway, the home physical activity environment, while acknowledging that it is one of several plausible, non-exclusive routes linking parental and child physical activity outcomes.

Despite this evidence base, several knowledge gaps persist. First, most existing research has focused on the direct association between parental physical activity and children’s activity levels rather than children’s physical fitness as a health outcome (15, 16). Physical fitness, which encompasses cardiorespiratory endurance, muscular strength, flexibility, and motor coordination, represents a more integrative indicator of physiological adaptation to habitual activity than physical activity volume alone (17).

Second, and more critically, the statistical pathways through which parental physical activity is associated with preschool children’s physical fitness remain poorly understood. Identifying these candidate mediating pathways is essential for designing targeted interventions, because understanding “how” a relationship works provides more actionable guidance than knowing merely that it exists (18).

The HPAE offers a theoretically compelling candidate for such a mediating mechanism. Bronfenbrenner’s bioecological model conceptualizes the family home as a primary microsystem (19, 20). Within this framework, proximal processes, understood as progressively complex reciprocal interactions between the developing child and the persons, objects, and symbols in the immediate environment, are theorized to drive developmental outcomes. More concretely, the physical attributes of the home, including the availability of sports equipment, the provision of indoor and outdoor activity space, family rules governing screen time and outdoor play, and parental behavioral support such as encouragement and co-participation, collectively constitute the “physical activity environment” that either facilitates or constrains children’s opportunities for active engagement (21, 22). Research by Maitland et al. (23) has demonstrated that the home physical environment, particularly the availability of activity equipment and the design of indoor space, influences children’s sedentary behavior and physical activity, although the evidence linking these environmental features directly to fitness outcomes remains limited. Sirard et al. (24) and Tandon et al. (25) further showed that parental rules and behavioral support within the home setting were associated with children’s physical activity and screen time, suggesting that the home environment functions as a multidimensional construct encompassing both tangible resources and social-regulatory practices.

Integrating these theoretical and empirical threads, the present study proposes that the HPAE statistically links parental physical activity and preschool children’s physical fitness. Specifically, physically active parents may be more likely to have more supportive home environments for movement, for example through purchasing more sports equipment, establishing pro-activity family rules, and providing greater behavioral support for their children’s physical engagement. These enriched home conditions, in turn, are hypothesized to be associated with greater physical activity opportunities and with superior physical fitness in preschool-aged children. This candidate pathway has not been empirically tested in the extant literature, particularly within the Chinese context where distinctive family structures, including the legacy of the one-child policy, the prevalence of multigenerational households, and strong cultural emphasis on academic achievement, may uniquely shape the home physical activity environment (26, 27).

The present study therefore pursued two objectives. The first was to examine the associations among parental physical activity, the home physical activity environment, and preschool children’s physical fitness in a sample of Chinese kindergarten families. The second was to test the statistical mediating role of the home physical activity environment in the association between parental physical activity and preschool children’s physical fitness. Based on the theoretical framework integrating social cognitive theory and Bronfenbrenner’s bioecological model, four hypotheses were formulated. H1 proposed a positive association between parental physical activity and preschool children’s physical fitness. H2 proposed a positive association between parental physical activity and the HPAE. H3 proposed a positive association between the home physical activity environment and preschool children’s physical fitness. H4 proposed that the HPAE would statistically mediate the association between parental physical activity and preschool children’s physical fitness.

## Materials and methods

### Study design and participants

This cross-sectional study was conducted between September and December 2025 in Nanchang, the capital city of Jiangxi Province, China. A multistage stratified random sampling strategy was employed. In the first stage, three administrative districts were selected from the six districts of Nanchang’s urban area to represent diverse socioeconomic profiles.

District selection was informed by 2024 government statistical yearbooks so that districts with contrasting per-capita income and urbanization levels were represented; the three districts were then chosen using a computer-generated random number list applied to the ranked list of eligible districts. In the second stage, 23 kindergartens (8 public and 15 private) were selected from the sampling frame of all registered kindergartens in the three districts using simple random sampling (computer-generated random numbers without replacement), stratified by public/private status to preserve the local public-private ratio. In the third stage, one to two classes per age group (3-year, 4-year, and 5-to-6-year classes) were selected within each kindergarten by simple random sampling (drawing lots from the full class roster of the relevant age group). All families within the selected classes were invited to participate.

Inclusion criteria required (a) the child to be aged 36–72 months at the time of testing, (b) the child to be in good health with no diagnosed motor impairment, chronic disease, or developmental disorder, and (c) at least one parent or primary caregiver to have provided informed consent and completed the survey questionnaire. Families were excluded if the child was absent on the day of physical fitness testing or if the questionnaire was incomplete.

A total of 612 parent–child dyads were initially recruited. After excluding 44 cases due to incomplete questionnaires (*n* = 28) or absent children on testing days (*n* = 16), 568 valid parent–child dyads were retained for analysis, yielding an effective response rate of 92.8%. The sample size exceeded the threshold of 462 recommended by Fritz and MacKinnon (28) for detecting indirect effects with small-to-medium path coefficients at 80% power. A post-hoc Monte Carlo simulation (2,000 replications, re-sampling the observed covariate distributions and simulating the mediator and outcome from the fitted model parameters) indicated that the achieved power to detect the observed indirect effect at *N* = 568 exceeded 0.99.

This study was approved by the Ethics Committee of Nanchang Vocational University (approval number NCVU-2025RT-2515). All participating parents provided written informed consent prior to data collection.

### Measures

#### Parental physical activity

Parental physical activity was assessed via the responding parent’s self-report using the Chinese version of the International Physical Activity Questionnaire-Short Form (IPAQ-SF). The IPAQ-SF asks respondents to report the frequency and duration of walking, moderate-intensity, and vigorous-intensity activity over the past 7 days without distinguishing the domain (occupational, transport-related, household, or leisure-time) in which the activity occurred; consequently, the present study characterizes overall habitual activity volume rather than activity within a specific life domain, a scope we revisit in the Limitations. Following the IPAQ-SF instructions, vigorous activities were defined as those that make the respondent breathe much harder than normal (e.g., heavy lifting, aerobics, running, or fast cycling), moderate activities as those that make the respondent breathe somewhat harder than normal (e.g., carrying light loads or cycling at a regular pace, excluding walking), and walking included walking at work, at home, for travel from place to place, and for recreation, sport, or leisure; only activities performed for at least 10 min at a time were counted. Weekday sitting time was also recorded but was not included in the MET computation. In the present sample, 63.0% of respondents were mothers and 37.0% were fathers. The IPAQ-SF has demonstrated acceptable reliability and validity in Chinese adult populations (29, 30) and captures the frequency (days per week) and duration (minutes per day) of walking, moderate-intensity, and vigorous-intensity physical activities performed over the past 7 days. Total physical activity was calculated as metabolic equivalent minutes per week (MET-min/week) following the standard IPAQ scoring protocol (31). Consistent with the official IPAQ scoring protocol, each reported activity duration was truncated at a maximum of 180 min per bout type prior to computing MET-min/week, and cases reporting more than 7 days per week for any activity type or a summed daily activity duration exceeding 960 min were flagged for exclusion as implausible; no cases in the present sample met these exclusion criteria (maximum reported daily durations were 67, 92, and 71 min for walking, moderate, and vigorous activity, respectively; maximum frequency was 7 days/week for walking and moderate activity and 4 days/week for vigorous activity), so no participants were removed at this screening stage. Because the distribution of total MET values exhibited positive skewness (skewness = 1.405), a natural logarithmic transformation, calculated as ln(1 + Total MET-min/week), was applied prior to regression analyses. Although this transformation reduced positive skewness, a residual left-skew persisted owing to a cluster of participants reporting zero physical activity (*n* = 23). Importantly, the bootstrap-based mediation test employed in this study (5,000 resamples) does not depend on the normality assumption for the sampling distribution of the indirect effect (32), rendering the results robust to this distributional departure. As an additional robustness check, we re-estimated the full mediation model after excluding the 23 zero-MET reporters; results are reported in the Results section and Supplementary sensitivity analyses.

#### Home physical activity environment

The HPAE was assessed using a purpose-designed questionnaire comprising 18 items organized into four dimensions. The item pool was initially generated by reviewing three established instruments for assessing the home physical activity environment in pediatric populations (24, 25, 33) and adapting their content to the Chinese preschool context. These three instruments were each developed for, and validated in, non-Chinese samples and settings that differ from urban Chinese preschool households in important respects (e.g., neighborhood-level walkability measures not readily applicable to high-density apartment living, and childcare-center rather than home-based item content); consequently, no single existing instrument provided adequate item coverage of all four dimensions targeted here, which motivated adaptation and the development of new items rather than direct adoption of an existing scale. Items were subsequently refined through a two-round Delphi consultation with five experts in early childhood physical education and pediatric public health, followed by cognitive interviews with 15 parents of preschool-aged children to ensure clarity and cultural appropriateness. A pilot study involving 80 parent–child dyads from two kindergartens not included in the main sample was conducted to assess preliminary reliability and to finalize item wording. The complete list of all 18 items with their Chinese and English versions is provided in Supplementary Table S2, including which of the three source instruments (or the Delphi/cognitive-interview process) informed the initial wording of each item; the complete item-level provenance mapping, indicating for each of the 18 items whether it was adapted from a source instrument or newly developed and the rationale for that decision, is provided in Supplementary Table S3. In summary, 11 of the 18 items were adapted from the three reviewed instruments (24, 25, 33), and 7 were newly developed through the Delphi and cognitive-interview process specifically to address features of urban Chinese preschool households not well captured by the source instruments (e.g., high-density apartment living, multigenerational co-residence, and locally salient safety concerns). The full Delphi rating sheets and cognitive-interview notes underlying Supplementary Table S3 are available from the corresponding author upon request. The Equipment Availability dimension (5 items) assessed the variety and sufficiency of physical activity equipment in the home (for example, “Our home has ball sports equipment such as soccer balls, basketballs, or badminton rackets”). The Activity Space dimension (4 items) assessed the availability of indoor and outdoor spaces for active play (for example, “There is enough indoor space at home for the child to run and jump”). The Family Rules dimension (4 items) assessed household regulations concerning screen time, outdoor play requirements, and sedentary behavior restrictions (for example, “We limit our child’s daily screen time on television and mobile devices”). The Behavioral Support dimension (5 items) assessed parental encouragement, co-participation in physical activity, transportation to sport programs, and active skill modeling (for example, “I often accompany my child in exercise or outdoor play”). Notably, this last item exemplifies a broader conceptual issue. Several Behavioral Support items describe parents’ own activity-related behavior rather than a fixed feature of the home setting, and may therefore overlap conceptually with parental physical activity itself. We address this overlap directly below and in a dedicated robustness check. All items were rated on a 5-point Likert scale ranging from 1 (strongly disagree) to 5 (strongly agree), with higher scores indicating a more supportive home physical activity environment.

The psychometric properties of the instrument were evaluated using the current sample, which is a limitation. Because scale development (including the confirmatory factor analysis reported below) and hypothesis testing were conducted in the same dataset, model-fit indices are likely optimistic relative to an independent sample, and the psychometric results below should be read as preliminary pending external cross-validation. Cronbach’s alpha coefficients were 0.862 for Equipment Availability, 0.834 for Activity Space, 0.802 for Family Rules, 0.878 for Behavioral Support, and 0.957 for the total scale. The total HPAE score was computed as the arithmetic mean of the four dimension subscale means (equal weighting of dimensions). Because the dimensions contain unequal numbers of items (5, 4, 4, and 5), this is not identical to the unweighted mean of the 18 individual items; the equal-dimension-weighting method was used consistently in all analyses, including the robustness check that excluded the Behavioral Support dimension (in which the total score was computed as the mean of the Equipment, Space, and Rules subscale means). All corrected item-total correlations (item vs. the sum of the remaining items in the same dimension) exceeded 0.30 (range 0.59–0.74). Confirmatory factor analysis showed close fit for the hypothesized four-factor model (*χ*^2^ = 151.85, df = 129, *χ*^2^/df = 1.177, CFI = 0.996, TLI = 0.996, RMSEA = 0.018 [90% CI 0.000, 0.028], SRMR = 0.018). All standardized factor loadings ranged from 0.66 to 0.79 (all *p* < 0.001). Composite reliability values exceeded 0.70 for all dimensions (range 0.801 to 0.879), and average variance extracted (AVE) values ranged from 0.502 to 0.592. The AVE for the Family Rules dimension (0.502) lay only at the conventional threshold of 0.50, which, together with the discriminant-validity results below, argues for interpreting this dimension cautiously. Importantly, the four-factor solution was not fully admissible, because three of the six standardized latent factor correlations slightly exceeded 1.0 (Equipment–Rules = 1.026, Space–Rules = 1.012, Rules–Support = 1.002; the remaining three ranged from 0.952 to 0.994) and the latent covariance matrix was therefore not positive definite. The four-factor model therefore cannot be taken as evidence of four distinct constructs. To provide a transparent basis for using the total score, Supplementary Table S4 presents the complete competing factor-model fit comparison. All CFA models were estimated with maximum likelihood in lavaan (34) using the same 18 items and the same sample, so their fit indices are directly comparable. The three-factor model (merging Equipment and Space; CFI = 0.995, RMSEA = 0.020; latent correlations again reaching 1.0), the two-factor model (tangible resources vs. social–regulatory practices; CFI = 0.994, RMSEA = 0.023; latent correlation = 0.996), and the single-factor model (CFI = 0.994, TLI = 0.993, RMSEA = 0.023, SRMR = 0.019; loadings 0.68 to 0.78) all showed close absolute fit. Chi-square difference tests nominally favored the four-factor model (Δ*χ*^2^ = 9.68, Δdf = 3, *p* = 0.021 for the three-factor model; Δ*χ*^2^ = 23.14, Δdf = 5, *p* < 0.001 for the two-factor model; Δ*χ*^2^ = 23.55, Δdf = 6, *p* < 0.001 for the single-factor model), but these differences were small, the practical fit indices were virtually identical across models (ΔCFI ≤ 0.002), and the comparison involves an improper four-factor solution. The near-equivalent fit of the single-factor model is consistent with a strongly saturated general factor. Near-unity correlations among factors do not by themselves establish unidimensionality, but together with the model-comparison and discriminant-validity results below they support treating the total score as a broad supportive-environment index rather than four independent constructs. To reduce the optimism that arises from developing and testing the scale in the same data, we additionally performed a split-sample cross-validation. The sample was randomly divided into two halves (*n* = 284 each). The single-factor model fitted closely in both halves (CFI = 0.997 and 0.996; RMSEA = 0.016 and 0.018; SRMR = 0.024 in each; standardized loadings 0.64–0.78 and 0.71–0.80), and a multigroup analysis supported configural, metric, and scalar invariance across the two halves (metric vs. configural, Δ*χ*^2^ = 8.71, Δdf = 17, *p* = 0.949; scalar vs. metric, Δ*χ*^2^ = 11.41, Δdf = 17, *p* = 0.835; no decrease in CFI). The primary mediation result was also replicated separately within each half (a × b = 0.091, 95% CI [0.058, 0.131], and a × b = 0.076, 95% CI [0.039, 0.118]; percentile bootstrap with 5,000 resamples). Because both halves are drawn from the same population, this procedure reduces, but does not replace, the need for validation in an independent sample. The complete bilingual version of this questionnaire is publicly available in Supplementary Table S2 to facilitate replication and cross-cultural adaptation by other research groups.

Because chi-square difference tests cannot by themselves establish that the four factors are practically distinct (and the four-factor solution was not fully admissible), we additionally evaluated discriminant validity using the Fornell-Larcker criterion (35) and the heterotrait-monotrait ratio of correlations (HTMT) Henseler et al. (36), as an additional check. Under the Fornell-Larcker criterion, the square root of each dimension’s AVE (Equipment = 0.746, Space = 0.746, Rules = 0.709, Support = 0.769) was consistently lower than the corresponding inter-dimension correlations (*r* = 0.817–0.866), indicating that the Fornell-Larcker criterion was not satisfied for any dimension pair. This failure, indicating substantial shared variance across dimensions, was further corroborated by the HTMT ratios (a more sensitive index recommended in recent methodological literature), which ranged from 0.95 to 1.02 across all six dimension pairs, well above the conservative 0.85 and even the liberal 0.90 threshold typically used to infer discriminant validity, and in several cases at or above 1.0. Taken together, these results indicate that although the four-factor model yields a nominally lower chi-square than more parsimonious alternatives, the four dimensions are not clearly empirically distinguishable from one another and most plausibly reflect a single, strongly saturated underlying construct, namely a broad supportive versus unsupportive home physical activity climate, rather than four independent facets. Accordingly, we treat the instrument as a preliminary measure, rely on the total score (not the four subscales) as the mediator in all primary analyses and avoid describing Equipment, Space, Rules, and Support as independent constructs. We report a corresponding robustness check that removes the conceptually overlapping Behavioral Support dimension from the total score; results were materially unchanged (see Results).

#### Preschoolers’ physical fitness

Preschoolers’ physical fitness was assessed using an adapted implementation based on the Chinese National Physical Fitness Measurement framework for preschool children (NPFM-PC; 《国民体质测定标准手册(幼儿部分)》), whose reliability has been established in previous research (37) and which has been employed in five national surveillance waves since 2000 (5). The term “battery” is used below in its standard psychometric sense (a coordinated set of subtests administered together) to refer to the specific set of tests as administered in this study; it does not imply that the present assessment is identical to the original surveillance protocol. The term NPFM-PC framework follows the terminology used in the primary methodological source (37) and other English-language reports of the Chinese National Physical Fitness Surveillance program; it is not an official English title of the Chinese instrument. The battery as administered in this study comprised six subtests, namely (1) sit-and-reach (坐位体前屈; flexibility; recorded in centimeters), (2) standing long jump (立定跳远; lower-limb explosive power; recorded in centimeters), (3) 10-meter shuttle run (10 米折返跑; agility; recorded in seconds), (4) tennis ball catching (网球接球; hand-eye coordination; the child stands 2 m from the tester and catches a softly tossed tennis ball with both hands; recorded as the number of successful catches out of 10 trials), (5) double-leg continuous jumping in 15 s (双脚连续跳 15 秒; lower-limb endurance; the child jumps continuously in place with feet together for 15 s; recorded as the number of completed jumps), and (6) sit-to-walk (坐姿起立行走; integrated locomotor ability; the child rises from a seated position on the floor, walks 3 m to a marked line, and returns to the seated position; recorded as total elapsed time in seconds). Higher counts indicate better performance on catching and continuous jumping; lower times indicate better performance on the shuttle run and sit-to-walk (the latter two were therefore reversed when forming the composite; see below).

These operationalizations adapt the classic NPFM-PC items described by Fang and Ho (37), which include tennis throwing, double-leg timed hop for a fixed number of hops, and balance-beam walking. Tennis ball catching was used to emphasize catching skill relevant to preschool play; continuous jumping was scored as repetitions in a fixed 15-s window rather than time to complete a fixed number of hops; and sit-to-walk served as a practical locomotor test feasible in the kindergarten setting. The catching subtest draws on the Movement Assessment Battery for Children-2 framework (38), although the MABC-2 does not directly establish reliability for the exact two-handed catching protocol used here. The sit-to-walk (Timed Up and Go) protocol has demonstrated acceptable test–retest reliability in 3- to 5-year-old children (39, 40). Continuous jumping scored by repetitions in a fixed time window, rather than by time to complete a fixed number of jumps, has been used as a field measure of lower-limb endurance in young children, although independent published reliability data for this exact 15-s variant are, to our knowledge, unavailable; this is noted as a limitation. Because three of the six subtests differ in format from the classic NPFM-PC version, the present battery should be understood as an adapted implementation of the NPFM-PC framework rather than an exact replication; however, the three retained subtests (sit-and-reach, standing long jump, and 10-m shuttle run) follow the standard NPFM-PC protocols precisely, and the three adapted subtests assess closely related motor capacities (coordination, lower-limb endurance, and locomotor ability). All tests were administered by trained research assistants following standardized protocols in kindergarten activity rooms or outdoor playgrounds.

To generate a composite fitness score that reduces age and sex differences, raw scores for each subtest were standardized within each of six subgroups defined by the crossing of three age groups (3-year, 4-year, and 5-to-6-year) and two sex categories (41) (subgroup sizes were 3-year boys *n* = 91, 3-year girls *n* = 89, 4-year boys *n* = 94, 4-year girls *n* = 83, 5-to-6-year boys *n* = 118, 5-to-6-year girls *n* = 93; standardization was performed within these six subgroups using only the final analytic sample of *N* = 568, i.e., after the exclusions described above, so that the *z*-scores reported throughout are internal to the analytic sample). For tests where lower times indicate better performance (10-meter shuttle run and sit-to-walk), *z*-scores were multiplied by −1 (reversed) before aggregation, so that higher values uniformly indicate better performance across all six subtests. The fitness composite was calculated as the arithmetic mean of the six age- and sex-standardized *z*-scores. Descriptive statistics for each fitness subtest stratified by age group and sex are provided in Supplementary Table S1. The effectiveness of this standardization procedure was confirmed by the near-zero correlations between the composite score and both age (*r* = 0.076) and sex (*r* = −0.000) after adjustment.

#### Covariates

Covariates included parental education level (coded 1 through 4 from junior high school or below to master’s degree or above) and monthly family income (coded 1 through 5 from below 3,000 RMB to above 20,000 RMB). These variables were selected on the basis of prior evidence indicating that socioeconomic status is associated with parental physical activity, the home environment, and children’s fitness (42, 43). Because the fitness composite was already standardized within age-sex subgroups, age and sex were not entered as covariates in the primary model; however, a supplementary analysis confirmed that their inclusion did not alter the results (see below).

The covariate strategy was further expanded for supplementary sensitivity analyses (reported fully in Results). Respondent gender and child BMI (*z*-standardized) were added to a first sensitivity model. Two further sets of covariates were added in additional sensitivity analyses. First, three child-level activity indicators were included to evaluate whether the parental-activity-home-environment-fitness pathway remains distinguishable from the child’s own behavior. These indicators, all reported by the same responding parent who completed the IPAQ-SF and home-environment questionnaire, were (a) the child’s average daily outdoor activity time (measured in minutes with the single item “Over the past 7 days, how many minutes per day on average did your child spend in outdoor play or physical activity?”, using a 7-day recall period), (b) weekly organized sport-session frequency (assessed with the single item “How many formal sport or structured physical-activity classes does your child attend per week?”; no fixed recall period; the response reflects current weekly enrollment), and (c) daily recreational screen time (measured in hours with the single item “On an average day, how many hours does your child spend on recreational television, mobile-device, or tablet use?”; no fixed recall period; the response reflects a typical day). Second, family structure (living with grandparents vs. not) and only-child status, to address the possibility that multigenerational household composition, which is common in urban China, confounds the associations under study.

In the primary mediation model, parental education and monthly family income were included as covariates because they are plausible common causes of parental physical activity, home-environment quality, and child fitness, and are temporally antecedent to all three focal variables. Child age and sex were not entered as covariates because the fitness composite was already standardized within age-sex subgroups (see Preschoolers’ physical fitness above); however, a supplementary analysis adding child age (in months) and child sex as covariates alongside parental education and family income confirmed that neither variable was significantly associated with the home-environment mediator (*β* = −0.041, *p* = 0.29 for age; *β* = 0.004, *p* = 0.75 for sex) or with the fitness composite after controlling for other covariates, and the mediation results were unchanged (indirect effect = 0.081, 95% CI [0.055, 0.109]).

Regarding kindergarten type (public vs. private), cluster-robust SEs address within-kindergarten dependence but do not control for confounding by an omitted kindergarten-level characteristic. We therefore conducted an additional sensitivity analysis in which kindergarten was entered as a fixed effect (22 dummy variables), thereby absorbing all time-invariant kindergarten-level characteristics, including but not limited to public/private status, resources, and neighborhood context. The results were essentially unchanged, with an indirect effect of 0.078 (95% bootstrap CI [0.051, 0.106]) compared with 0.081 (95% CI [0.055, 0.109]) in the primary model. This analysis suggests that the primary mediation estimate is robust to adjustment for kindergarten-specific fixed effects, suggesting that kindergarten-level heterogeneity is unlikely to fully account for the observed indirect association. Child-level behavioral variables (outdoor activity time, organized sport participation, and screen time) were included in a separate sensitivity model. Their causal position relative to parental activity and the home environment cannot be determined from these cross-sectional data: they could be intermediate variables, confounders, or correlates of shared family influences. The primary and child-activity-adjusted models therefore describe associations conditional on different covariate sets and are interpreted together.

### Statistical analysis

The statistical methods applied in this study were (1) descriptive statistics (means/SDs for continuous variables; frequencies/percentages for categorical variables); (2) Pearson correlations for bivariate associations among study variables (Table 1); (3) confirmatory factor analysis (lavaan) for the home physical activity environment scale, including composite reliability, average variance extracted, Fornell-Larcker, and HTMT discriminant-validity indices; (4) Harman’s single-factor test and a correlational marker-variable adjustment (44) for common method bias; (5) variance inflation factor checks for multicollinearity; (6) the primary hypothesis test, a PROCESS Model 4 bootstrap mediation analysis (5,000 resamples) for parental physical activity → home physical activity environment → preschool children’s fitness; (7) OLS regression (Python/statsmodels) to obtain unstandardized coefficients and to conduct all supplementary sensitivity analyses (excluding zero-MET reporters; adding child activity/screen-time covariates; adding family-structure covariates; removing the Behavioral Support dimension) and moderated-mediation (interaction-term) tests; and (8) a Monte Carlo simulation-based post-hoc power estimate. Full detail on each method is given at its point of first use below and is cross-referenced from the Results.

**Table 1:** Means, standard deviations, and Pearson correlations among key study variables (*N* = 568).

Variable	1	2	3	4	5	6	7
1. ln(total MET)	–						
2. Equipment	0.349^***^	–					
3. Space	0.316^***^	0.834^***^	–				
4. Rules	0.337^***^	0.853^***^	0.827^***^	–			
5. Behavioral support	0.322^***^	0.866^***^	0.817^***^	0.841^***^	–		
6. HPAE total	0.353^***^	0.947^***^	0.927^***^	0.937^***^	0.940^***^	–	
7. Fitness composite	0.227^***^	0.343^***^	0.343^***^	0.332^***^	0.323^***^	0.357^***^	–
*M*	6.19	2.99	3.20	3.06	3.43	3.17	0.000
SD	1.65	0.77	0.76	0.75	0.78	0.72	0.734

^***^p < 0.001. *N* = 568. HPAE, home physical activity environment.

All analyses were performed using R version 4.3.1 (with the lavaan package version 0.6–16 for CFA) and SPSS 26.0 with the PROCESS macro version 4.1, with supplementary sensitivity and robustness analyses (described below) conducted in Python 3.11 (pandas, statsmodels, numpy) for full reproducibility. Visual inspection of residual plots confirmed that assumptions of linearity and homoscedasticity were adequately met for all regression models. Descriptive statistics were computed for all study variables (means and standard deviations for continuous variables; frequencies and percentages for categorical variables). Pearson correlation coefficients were calculated to examine bivariate associations among the key variables. Prior to mediation analysis, assumptions of normality, linearity, and multicollinearity were evaluated. Variance inflation factors for all predictors were below 5.0, indicating acceptable multicollinearity.

Mediation analysis followed the approach outlined by Hayes (45) using Model 4 with 5,000 bootstrap resamples. The independent variable was log-transformed parental physical activity (ln_Total_MET), the mediator was the HPAE total score, and the dependent variable was the age- and sex-standardized fitness composite. The primary model included parental education and family income as covariates. The child-activity-adjusted sensitivity model additionally included children’s outdoor activity time, organized sport participation, and recreational screen time as additive covariates in both the mediator and outcome equations. The indirect effect was considered statistically significant if the 95% bias-corrected bootstrap confidence interval did not include zero (32).

Because participants were drawn from 23 kindergartens, the potential for clustered data was evaluated by computing the intraclass correlation coefficient (ICC) for the fitness composite. The ICC for the age-sex-standardized fitness composite was 0.002 (Table 2, Panel A; computed using the verified kindergarten identifier), indicating that between-kindergarten variance was limited relative to within-kindergarten variance and implying only minimal inflation of the sampling variance due to kindergarten-level clustering (design effect ≈ 1.05). This result suggests that kindergarten-level clustering had limited influence on the outcome variable, likely because fitness scores were already standardized within age-sex subgroups, absorbing much of the between-kindergarten variance attributable to compositional differences. Accordingly, single-level regression was considered appropriate for the primary analysis. Because age-sex standardization may reduce between-kindergarten variance associated with age and sex composition, a near-zero ICC for the standardized composite alone cannot rule out kindergarten-level clustering. Three supplementary assessments used kindergarten identifiers for all 568 participants. First, we computed the ICC on each of the six fitness subtests in their fully raw, unstandardized units (i.e., prior to any age-sex adjustment), so that these estimates did not depend on age-sex standardization. Raw ICCs were 0.000 (sit-and-reach; the estimate was slightly negative, −0.001, and is reported as 0.000), 0.007 (standing long jump), 0.002 (10-m shuttle run), 0.004 (ball catching), 0.008 (continuous jumping), and 0.012 (sit-to-walk), all likewise negligible, indicating that kindergarten membership explains essentially none of the variance in fitness performance even before any standardization is applied. Second, we recomputed the ICC for the age-sex-standardized composite directly using the kindergarten identifier (ICC = 0.002), confirming a near-zero value. Third, we re-estimated the primary mediation model (Table 3, Steps 1–3) using kindergarten-clustered robust (sandwich-estimator) standard errors and, because only 23 clusters were available, additionally ran a cluster-level bootstrap (3,000 resamples of kindergartens with replacement) for the indirect effect. All paths remained significant under clustering, with cluster-robust standard errors of similar or smaller magnitude than the conventional OLS standard errors (*β* = 0.349, cluster-robust SE = 0.033, *p* < 0.001 for path a; *β* = 0.234, cluster-robust SE = 0.019, *p* < 0.001 for path b; *β* = 0.167, cluster-robust SE = 0.029, *p* < 0.001 for path c; *β* = 0.085, cluster-robust SE = 0.025, *p* < 0.001 for path c′), and the cluster-bootstrap 95% CI for the indirect effect, [0.060, 0.104], closely matched and remained fully consistent with the original individual-level bootstrap CI of [0.055, 0.109]. Taken together, the raw-score ICCs and cluster-adjusted analyses support the use of single-level inference for the primary analyses (see Table 2 for the complete ICC and cluster-robust results). All ICCs were computed by one-way random-effects ANOVA using each child’s verified kindergarten identifier; the full ANOVA output underlying each ICC (between- and within-kindergarten mean squares, F-statistics, and degrees of freedom) is reported in Supplementary Table S6.

**Table 2:** Kindergarten-clustering diagnostics and cluster-robust re-analysis of the primary mediation model.

Panel A. Intraclass correlation coefficients	
Fitness subtest	ICC
Sit-and-reach (cm)	0.000ᵃ
Standing long jump (cm)	0.007
10-m shuttle run (s)	0.002
Tennis ball catching (count)	0.004
Continuous jumping/15 s (count)	0.008
Sit-to-walk (s)	0.012
Fitness composite (*z*-score)	0.002
ICCs computed by one-way random-effects ANOVA as ICC(1) = (MSB−MSW)/(MSB + (*n*₀ − 1) × MSW), where MSB = between-kindergarten mean square, MSW = within-kindergarten mean square, and *n*₀ is the average cluster size. Because kindergarten sizes were unequal (range 12–34), *n*₀ was set to the arithmetic mean (*N*/*K* = 568/23 = 24.7). Using instead the unequal-cluster-size adjusted value, *n*₀ = [*N* − *Σnj*^2^/*N*]/(*K* − 1) = 24.64, yields identical ICCs to three decimal places. All subtest ICCs were computed on raw (unstandardized) scores; the composite ICC used the age-sex-standardized *z*-scores. All values were computed using each child’s verified kindergarten identifier as the grouping variable (*K* = 23, *N* = 568). ᵃThe sit-and-reach estimate was slightly negative (−0.001; *F* < 1) and is reported as 0.000. Full ANOVA output (MSB, MSW, *F*-statistics, and degrees of freedom for each subtest and the composite) is provided in Supplementary Table S6.	

Panel B. Cluster-robust re-estimation of the primary mediation model				
Path	*β* (OLS)	Conventional SE	Cluster-robust SE	Cluster-robust *p*
c (total)	0.167	0.030	0.029	<0.001
a	0.349	0.039	0.033	<0.001
b	0.234	0.031	0.019	<0.001
c′ (direct)	0.085	0.031	0.025	<0.001
Coefficients and conventional SEs are identical to those in Table 3 because both tables derive from the same estimation specification (same covariates, covariate coding, and standardization of focal variables). Cluster-robust (sandwich-estimator) SEs computed with kindergarten as the clustering variable (23 clusters). A cluster-level bootstrap (3,000 resamples of kindergartens with replacement) for the indirect effect yielded a × b = 0.081, 95% CI [0.060, 0.104], closely matching the individual-level bootstrap CI reported in Table 3 ([0.055, 0.109]).				

**Table 3:** Results of the primary mediation analysis using PROCESS Model 4 (*N* = 568).

Path	Description	*β*	*B*	SE	*t*	*p*	95% CI	*R* ^2^
c (total)	PA → Fitness	0.167	0.101	0.030/0.018	5.53	<0.001		0.055
a	PA → HPAE	0.349	0.151	0.039/0.017	8.87	<0.001		0.132
b	HPAE → Fitness	0.234	0.326	0.031/0.043	7.59	<0.001		
c′ (direct)	PA → Fitness adj.	0.085	0.052	0.031/0.019	2.78	0.006		0.142
a × b (indirect)		0.081	0.049				[0.055, 0.109]	

*β* coefficients are partially standardized, meaning that the predictor (ln-MET, SD = 1.65) and mediator (HPAE total, SD = 0.72) were standardized to unit variance, but the fitness composite was retained in its native age-sex-standardized *z*-score units (SD = 0.734). Consequently, *β* = *B* × SD (predictor-in-that-path), that is, for paths c and c′, *β* = *B* × 1.65; for path a, *β* = *B* × 1.65/0.72; for path b, *β* = *B* × 0.72. For each path, the SE column reports SE(*β*)/SE(*B*), where the first value is the standard error of the partially standardized *β*, and the second is the standard error of the fully unstandardized *B*. Bootstrap = 5,000 resamples. Covariates: parental education, family income.

Statistical significance was set at *p* < 0.05 (two-tailed). Common method bias was evaluated using Harman’s single-factor test (46); the first unrotated factor accounted for 28.6% of the total variance, below the 40% threshold indicating problematic shared method variance.

Because parental physical activity and the home physical activity environment were both reported by the same responding parent on the same occasion, Harman’s single-factor test, which evaluates shared variance across all measured constructs simultaneously, is a relatively insensitive check for method bias that is concentrated in two specific constructs. Harman’s test therefore cannot rule out common-method inflation of the parental-activity→home-environment path (path a). As a supplementary check, we applied the correlational marker technique of Lindell and Whitney (44). Because no purpose-designed marker scale was included in the survey, parental age, which was reported by the same respondent on the same occasion and is theoretically unrelated to the home environment, served as the marker variable. Its correlations with ln-MET (*r* = −0.052) and with the HPAE total score (*r* = −0.032) provided the estimate of method variance, and the larger absolute value (*rM* = 0.052) was used as a conservative choice. The method-adjusted correlation between parental physical activity and the home environment was 0.318 (unadjusted *r* = 0.353; *t* = 7.97, *p* < 0.001). Path a would lose statistical significance only if method variance accounted for a correlation of about 0.30, that is, nearly all of the observed association. With the adjusted correlation, the covariate-free indirect effect decreased only modestly (from 0.112 to 0.101). Because parental age is a demographic report rather than a perceptual rating, it is an imperfect marker, and this check should be read as supportive rather than conclusive; a purpose-designed marker scale is recommended for future data collection.

## Results

### Participant characteristics

The demographic and descriptive characteristics of the sample are presented in Table 4. Of the 568 participating children, 303 (53.3%) were boys and 265 (46.7%) were girls. The mean age was 54.6 months (SD = 10.9), distributed across three age groups with 180 in the 3-year group, 177 in the 4-year group, and 211 in the 5-to-6-year group. The mean BMI was 16.09 kg/m^2^ (SD = 3.34). Among the respondent parents, 358 (63.0%) were mothers and 210 (37.0%) were fathers, with a mean age of 33.0 years (SD = 3.9). The majority of parents held associate or bachelor’s degrees (48.1%), and the most common income bracket was 5,000–10,000 RMB per month (38.9%).

**Table 4:** Participant characteristics (*N* = 568).

Variable	*n* or *M*	% or SD
Child characteristics		
Sex (male/female)	303/265	53.3/46.7
Age (months)	54.6	10.9
3-year group	180	31.7
4-year group	177	31.2
5-to-6-year group	211	37.1
Height (cm)	104.3	9.6
Weight (kg)	17.3	3.0
BMI (kg/m^2^)	16.09	3.34
Parent characteristics		
Respondent (father/mother)	210/358	37.0/63.0
Age (years)	33.0	3.9
Education		
Junior high school or below	39	6.9
Senior high school/vocational	132	23.2
Associate/bachelor degree	273	48.1
Master degree or above	124	21.8
Monthly family income (RMB)		
<3,000	42	7.4
3,000–5,000	87	15.3
5,000–10,000	221	38.9
10,000–20,000	161	28.3
>20,000	57	10.0
Family structure (nuclear/grandparent/other)	227/283/58	40.0/49.8/10.2
Only child (yes/no)	201/367	35.4/64.6
Parental physical activity		
Total MET (MET-min/week)	932.3	823.0
ln(1 + Total MET)	6.19	1.65
PA level (low/moderate/high)	254/301/13	44.7/53.0/2.3
Daily sitting time (hours)	6.5	1.6
Home PA environment (1–5 scale)		
Equipment availability	2.99	0.77
Activity space	3.20	0.76
Family rules	3.06	0.75
Behavioral support	3.43	0.78
Total score	3.17	0.72
Child physical activity behaviors		
Outdoor activity time (min/day)	58.3	27.0
Organized sport sessions (per week)	1.34	1.06
Recreational screen time (hours/day)	1.98	0.69
Preschool children’s physical fitness		
Sit-and-reach (cm)	11.98	3.20
Standing long jump (cm)	89.28	17.60
10-m shuttle run (s)	7.60	0.95
Tennis ball catching (count)	4.89	1.99
Continuous jumping/15 s (count)	21.13	5.21
Sit-to-walk (s)	5.48	0.69
Fitness composite (*z*-score)	0.000	0.734

Continuous variables as *M* (SD); categorical as *n* (%). PA, physical activity; HPAE, home physical activity environment.

Regarding parental physical activity, the mean total MET was 932.3 MET-min/week (SD = 823.0), with a median of 690.0 MET-min/week. After log transformation, the skewness was reduced from 1.41 (raw Total MET) to an acceptable level for regression, and the bootstrap procedure further ensured robust inference regardless of residual non-normality. According to the IPAQ categorical scoring, 254 parents (44.7%) were classified as having low physical activity levels, 301 (53.0%) as moderate, and 13 (2.3%) as high. Parents reported an average of 6.5 h (SD = 1.6) of daily sitting time.

The HPAE total score averaged 3.17 (SD = 0.72) on the 5-point scale. Among the four dimensions, Behavioral Support received the highest mean rating (*M* = 3.43, SD = 0.78), followed by Activity Space (*M* = 3.20, SD = 0.76), Family Rules (*M* = 3.06, SD = 0.75), and Equipment Availability (*M* = 2.99, SD = 0.77).

For preschool children’s physical fitness, the mean standing long jump distance was 89.28 cm (SD = 17.60), the mean sit-and-reach distance was 11.98 cm (SD = 3.20), and the mean 10-meter shuttle run time was 7.60 s (SD = 0.95). The age- and sex-standardized fitness composite had a mean of 0.000 (SD = 0.734), indicating successful age- and sex-standardization of the fitness composite.

### Correlation analysis

Table 1 presents the Pearson correlation coefficients among the key study variables. Log-transformed parental physical activity (ln_Total_MET) was significantly positively correlated with all four dimensions of the HPAE, with coefficients ranging from *r* = 0.316 (Activity Space) to *r* = 0.349 (Equipment Availability), and with the total home environment score (*r* = 0.353, *p* < 0.001). Parental physical activity was also positively correlated with the fitness composite (*r* = 0.227, *p* < 0.001). The home physical activity environment total score was positively correlated with the fitness composite (*r* = 0.357, *p* < 0.001), with the four dimensions showing similar correlations with fitness (*r* = 0.323 to 0.343, all *p* < 0.001). These patterns of bivariate associations were consistent with the prerequisites for mediation analysis, as significant correlations were observed among all three focal variables (47).

As detailed above and in Table 1, the inter-dimension correlations (*r* = 0.817–0.866) and the HTMT ratios (0.95–1.02) indicate that the four home-environment dimensions are not empirically well separated; this pattern, together with the correlations in Table 1, is discussed further under Discriminant validity above and in the Discussion.

### Mediation analysis

Table 3 and Figure 1 present the results of the mediation analysis. The primary model controlled for parental education and family income as covariates. In Step 1, regressing the fitness composite on log-transformed parental physical activity (with covariates) yielded a significant total association (path c, *β* = 0.167, SE = 0.030, *t* = 5.53, *p* < 0.001, *R*^2^ = 0.055). In Step 2, regressing the home physical activity environment on parental physical activity yielded a significant path a association (*β* = 0.349, SE = 0.039, *t* = 8.87, *p* < 0.001, *R*^2^ = 0.132). In Step 3, when both parental physical activity and the home physical activity environment were entered simultaneously as predictors of the fitness composite, the home environment was significantly associated with fitness (path b, *β* = 0.234, SE = 0.031, *t* = 7.59, *p* < 0.001), and the direct association of parental physical activity was attenuated but remained significant (path c′, *β* = 0.085, SE = 0.031, *t* = 2.78, *p* = 0.006). The full model explained 14.2% of the variance in preschool children’s fitness (*R*^2^ = 0.142).

**Figure 1:**
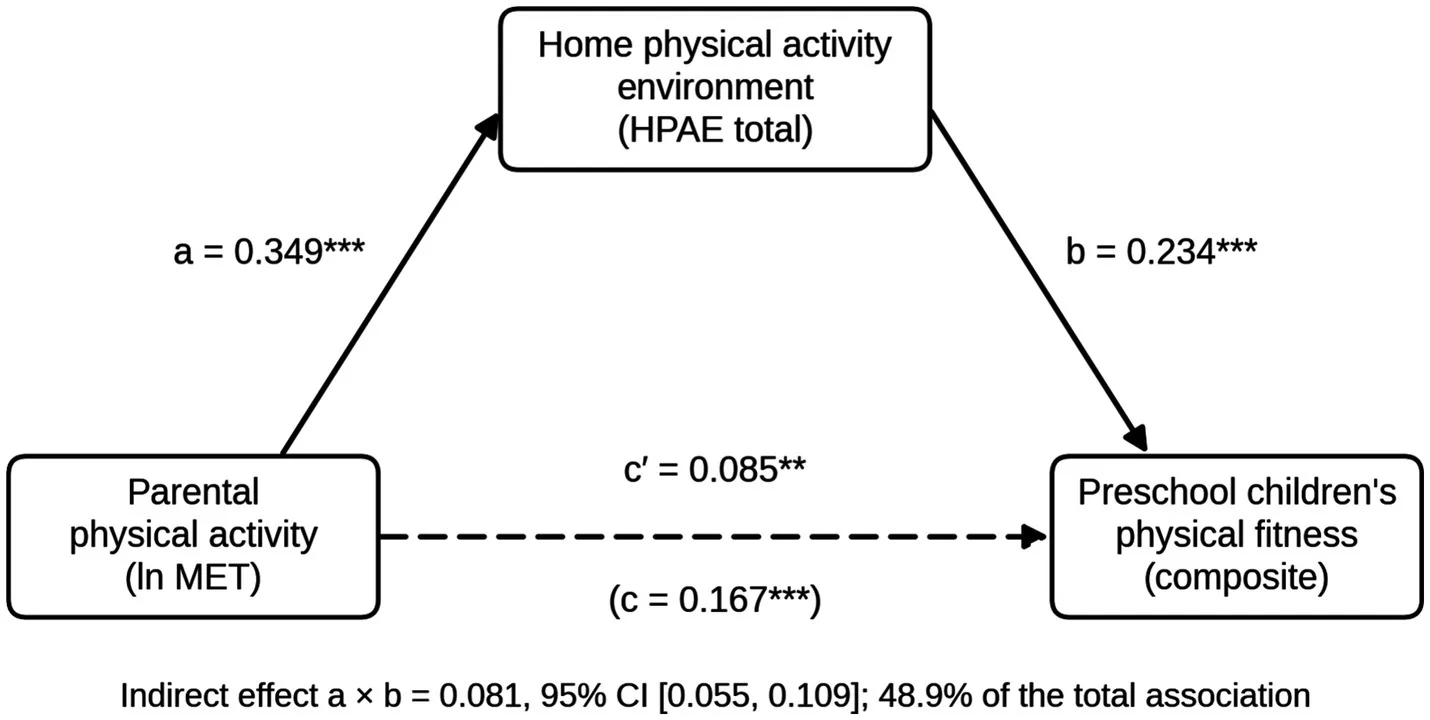
Primary cross-sectional statistical mediation model depicting the associations among parental physical activity, the home physical activity environment, and preschool children’s physical fitness (*N* = 568). Partially standardized path coefficients are shown (see Table 3 note for definition). Solid lines represent significant paths in the mediation model; the dashed line represents the direct path after controlling for the mediator. The model was adjusted for parental education and family income. The bootstrap-based indirect association was a × b = 0.081 (95% CI [0.055, 0.109]). The 48.9% mediated proportion applies only to this primary model. The model does not establish temporal or causal ordering. After additional adjustment for children’s outdoor activity, organized sport participation, and screen time, the indirect association was close to zero and was not statistically significant (a × b = −0.002, 95% CI [−0.013, 0.007]; Table 5 and Supplementary Table S5). ^**^p < 0.01, ^***^p < 0.001.

Unstandardized coefficients are also reported alongside the standardized values in Table 3 to aid practical interpretation. In raw units, a one-unit increase in log-transformed parental MET-min/week (ln_Total_MET) was associated with a 0.151-point increase (on the 1–5 scale) in the HPAE score (*B* = 0.151, SE = 0.017, *p* < 0.001), and a one-point increase in the home environment score was associated with a 0.326-point increase in the fitness composite (*B* = 0.326, SE = 0.043, *p* < 0.001), holding covariates constant. Equivalently, a one-SD difference in ln_Total_MET (SD = 1.65) corresponds to about a 0.25-point difference in home environment score, and a one-SD difference in the home environment score (SD = 0.72) corresponds to about a 0.23-point difference in the fitness composite, which is a small-to-moderate effect in the context of a composite index with SD = 0.73 in this sample.

The bootstrap analysis revealed a significant indirect effect (a × b = 0.081, SE = 0.013, 95% CI [0.055, 0.109]). Because the confidence interval did not contain zero, the primary model was consistent with the indirect association hypothesized in H4. The unstandardized indirect effect (*B* = 0.049) was identical to the difference between the total and direct effects (*B* = 0.101–0.052 = 0.049), confirming internal consistency of the mediation decomposition. The 48.9% mediated proportion (a × b/c) applies only to the primary model and describes a statistical decomposition of the observed association. Neither this proportion nor the significant direct association establishes a causal mediation mechanism or identifies other causal pathways. In the child-activity-adjusted model described below, the indirect association was close to zero and was not statistically significant.

Figure 1 depicts the path coefficients from the primary (two-covariate) model. A supplementary sensitivity analysis was conducted to evaluate the robustness of the mediation findings to the inclusion of additional covariates. Respondent gender (1 = father, 2 = mother) and child BMI (*z*-standardized) were added to the model alongside parental education and family income. The results remained stable compared to the primary model, with minimal changes in path coefficients (Δ*β* < 0.005 for all paths) (path c, *β* = 0.167; path a, *β* = 0.348; path b, *β* = 0.234; indirect effect a × b = 0.081, 95% CI [0.055, 0.109]; mediation proportion = 48.8%), suggesting that the observed indirect association was not materially altered by adjustment for respondent gender or child adiposity.

### Additional sensitivity, robustness, and moderation analyses

Five further sensitivity, robustness, and moderation analyses were conducted on the full dataset and are summarized in Table 5 and narratively below.

**Table 5:** Supplementary sensitivity, robustness, and moderation analyses.

Analysis	Indirect *β*	95% CI	Notes
Primary model (Table 3)	0.081	[0.055, 0.109]	Covariates: education, income
+ Respondent gender and child BMI	0.081	[0.055, 0.109]	Negligible change
+ Child outdoor, sport, screen	−0.002	[−0.013, 0.007]	CI includes zero
+ Family structure and only-child	0.081	[0.055, 0.109]	Negligible change
Excl. zero-MET reporters (*n* = 545)	0.064	[0.042, 0.089]	Attenuated, still significant
Excl. Behavioral Support dim.	0.082	[0.056, 0.110]	Proportion = 49.4%
Moderation by respondent gender			Index = −0.013, CI [−0.075, 0.053]
Moderation by child age group			Index = 0.019, CI [−0.044, 0.079]

All indirect effects from bootstrap with 5,000 resamples. CI = 95% bias-corrected confidence interval.

First, excluding the 23 participants who reported zero MET-min/week (a residual left-skew in the log-transformed variable noted above) yielded a somewhat attenuated but still significant indirect effect (*n* = 545; path a, *β* = 0.287, *p* < 0.001; path b, *β* = 0.222, *p* < 0.001; a × b = 0.064, 95% CI [0.042, 0.089]; mediation proportion = 41.0%), indicating that the primary-model indirect association is not driven solely by this subgroup, although the somewhat smaller proportion suggests the zero-reporters contribute non-trivially to the estimated association and warrants mention as a limitation of self-reported activity data.

Second, and most substantively, we added three child-level covariates, namely the child’s average daily outdoor activity time, weekly organized sport-session frequency, and daily recreational screen time, to the primary model, to assess how the estimated indirect association changed after accounting for these behaviors, whose causal position cannot be established from the cross-sectional data. Child outdoor activity time correlated moderately-to-strongly with all three focal variables in this sample (*r* = 0.395 with parental ln-MET, *r* = 0.597 with the home environment total score, and *r* = 0.421 with the fitness composite). When these three child-level variables were added as covariates, the model’s explained variance in fitness rose sharply (*R*^2^ = 0.203, versus 0.142 in the primary model), the a-path (parental activity → home environment) became non-significant (*β* = −0.015, *p* = 0.66), and the indirect effect through the home environment was no longer distinguishable from zero. With the three child-level variables entered as additive covariates in both the mediator (a-path) and outcome (b-path) equations, the complete estimates were *β* = −0.015 (SE = 0.034, *t* = −0.44, *p* = 0.661) for path a, *β* = 0.153 (SE = 0.040, *t* = 3.81, *p* < 0.001) for path b, and *β* = 0.057 (SE = 0.032, *t* = 1.76, *p* = 0.079) for path c′, with model *R*^2^ = 0.203 (Supplementary Table S5 presents the full regression output for this sensitivity analysis). The indirect effect was a × b = −0.002 (95% CI [−0.013, 0.007]). Thus, after additional adjustment for children’s outdoor activity, organized sport participation, and screen time, the estimated indirect association was close to zero and was not statistically significant. The indirect association observed in the primary model was therefore not robust to inclusion of these child-level variables.

Third, adding family structure (living with grandparents, coded from 227 nuclear, 283 grandparent-coresident, and 58 other households) and only-child status (201 only children, 367 with siblings) as covariates left the primary results essentially unchanged (path a, *β* = 0.348; path b, *β* = 0.234; a × b = 0.081, 95% CI [0.055, 0.109]; mediation proportion = 49.0%), suggesting that multigenerational household composition and sibling status, while important to acknowledge conceptually, do not appear to confound the primary association in this sample.

Fourth, because the Behavioral Support dimension (e.g., “I often accompany my child in exercise or outdoor play”) may conceptually overlap with parental physical activity itself rather than purely reflect a home-environment feature, we re-ran the mediation model using a reduced home-environment score computed from only the Equipment, Space, and Rules dimensions. Results were materially unchanged (path a, *β* = 0.349; path b, *β* = 0.235; a × b = 0.082, 95% CI [0.056, 0.110]; mediation proportion = 49.4%), indicating that the indirect association is not driven solely by the Behavioral Support dimension.

Fifth, we tested moderated mediation by respondent gender (father vs. mother) and by child age group, to examine potential moderating effects. Neither the a-path nor the b-path was significantly moderated by respondent gender (interaction *β* = −0.071, *p* = 0.37 for path a; *β* = 0.045, *p* = 0.46 for path b) or by child age group (interaction *β* = −0.019, *p* = 0.67 for path a; *β* = −0.030, *p* = 0.39 for path b). These non-significant interaction tests do not establish the absence of moderation, because their statistical power may be limited relative to main-effect tests even at *N* = 568. Larger, purpose-designed studies are needed to examine potential moderation.

## Discussion

This study examined cross-sectional associations among parental physical activity, the home physical activity environment, and preschool children’s physical fitness among 568 Chinese kindergarten families. In the primary model, adjusted for parental education and family income, the observed associations were consistent with the hypothesized potential pathway through the home environment. However, the indirect association was reduced to approximately zero after adjustment for children’s outdoor activity, organized sport participation, and screen time (a × b = −0.002, 95% CI [−0.013, 0.007]) and was not statistically significant. The findings therefore describe a model-dependent pattern of associations rather than establish a mediation mechanism.

### Parental physical activity and preschool children’s physical fitness

The finding that parental physical activity was positively associated with preschool children’s physical fitness (H1) extends prior work that has predominantly examined parent–child concordance in activity levels rather than fitness outcomes. The total association estimated in the primary model (*β* = 0.167) was small in magnitude, aligning with the “weak positive” associations reported by Petersen et al. (11) across 39 studies. The present study examined parental activity in relation to a composite of children’s fitness-test performance, extending the focus beyond concordance in reported activity behavior (17). This association does not demonstrate a parental influence on children’s fitness. This distinction matters because physical fitness in early childhood has been shown to predict cardiometabolic risk profiles in later life independently of concurrent physical activity levels (48, 49).

The relatively modest size of the total association in the primary model should be interpreted in light of the multifactorial nature of preschool children’s physical fitness, which is shaped by genetic endowment, nutritional status, kindergarten programming, and maturational tempo in addition to family influences (50, 51). Consistent with this multifactorial view, when we additionally accounted for the child’s own outdoor activity time, sport-session frequency, and screen time in a supplementary analysis (see Results), the explained variance in fitness increased substantially (*R*^2^ = 0.203) and the parental-activity → home-environment path became statistically indistinguishable from zero. The indirect association was also close to zero and not statistically significant in that adjusted model. The 48.9% mediated proportion applies only to the primary model and cannot establish the effectiveness of modifying family-level factors. Prior research provides a rationale for investigating these factors: Schoeppe et al. (52) reported that both maternal and paternal physical activity modeling were independently associated with children’s leisure-time physical activity, and Jago et al. (53) reported that physical activity-based parenting practices mediated the link between nurturant parenting styles and young children’s moderate-to-vigorous physical activity.

### The home physical activity environment as a potential pathway

In the primary model, the significant indirect association through the HPAE was consistent with the potential pathway hypothesized in H4. The 48.9% mediated proportion applies only to that model. It is a descriptive decomposition of the observed cross-sectional association, not evidence that nearly half of a causal parental effect is transmitted through the home environment. After additional adjustment for children’s outdoor activity, organized sport participation, and screen time, the indirect association was close to zero and was not statistically significant. Considered together, the two models indicate that the proposed pathway is sensitive to covariate specification and do not provide robust evidence of mediation.

The associations in the primary model are compatible with Bronfenbrenner’s bioecological model (19), which provides a theoretical rationale for examining the home microsystem in relation to parental and child characteristics. However, theoretical compatibility does not distinguish this proposed pathway from alternative explanations, particularly given the near-zero indirect association in the child-activity-adjusted model. Physically active parents tend to report more sports equipment, rules that limit sedentary time and promote outdoor play, and greater co-participation in physical activities with their children. These environmental features may provide affordances (54) that are associated with children moving, exploring, and developing their physical capacities, although the present design cannot establish this as a causal sequence.

The finding also refines existing evidence on the home physical environment and children’s activity behaviors. Maitland et al. (23) noted that home physical activity equipment was inversely associated with sedentary behaviors in children but showed inconsistent associations with physical activity, partly because of measurement limitations. The present study addressed some of these limitations by employing a multidimensional measure of the home environment that captured not only tangible equipment but also spatial access, regulatory practices, and social support.

However, as detailed in Methods and Results, the four dimensions of this measure were not empirically well separated (HTMT ratios of 0.95–1.02), so this multidimensionality should currently be regarded as conceptual rather than empirically confirmed; the indirect association estimated in the primary model concerns a broad supportive-environment index and does not identify four independent mediating components.

In the mediation model, the HPAE was entered as a composite total score rather than as separate dimensions, given the comparable correlation magnitudes observed across all four dimensions (*r* = 0.323–0.343 with the fitness composite; see Table 1) and, as elaborated above, given the weak empirical separability of the four dimensions. Moreover, because the inter-dimension correlations were very high (*r* = 0.817–0.866; Table 1), entering the four dimensions simultaneously as parallel mediators would introduce severe multicollinearity, so dimension-specific indirect effects cannot be meaningfully disaggregated in these data.

The significant direct association in the primary model does not identify the processes underlying the remaining parental activity–fitness association. Potential explanations include shared genetic influences (14), parental modeling outside the home (55), and shared family routines or resources. The near-zero indirect association after adjustment for children’s outdoor activity, organized sport participation, and screen time is particularly important for interpretation. One possibility is that child activity lies downstream of the home environment; conditioning on an intermediate variable would then change the association being estimated. Another possibility is that child activity or shared family influences confound the associations among parental activity, the home environment, and fitness. These are alternative hypotheses, not explanations established by the present analyses. The adjusted result cannot be dismissed as over-control, nor can it establish the absence of every possible home-environment pathway. It does show that the primary-model indirect association is not robust to this covariate specification. Longitudinal measurements with clear temporal ordering and experimental studies are needed to distinguish these possibilities.

### Alternative explanations

Beyond the proposed pathway, several alternative explanations are equally consistent with the present cross-sectional correlations and cannot be ruled out by the mediation model as specified. First, reverse or child-driven pathways are plausible. Children who are constitutionally fitter, more motorically skilled, or more movement-oriented may elicit more parental encouragement, more equipment purchases, and more structured activity routines at home, rather than the home environment causing fitness. Second, common-cause explanations are plausible. Families with greater health consciousness, health literacy, or socioeconomic resources may simultaneously have more physically active parents, more supportive home environments, and fitter children, without either parental activity or the home environment exerting a causal effect on fitness net of this shared background factor; our finding that parental education and family income were retained as covariates addresses this only partially, since unmeasured aspects of health consciousness and resource availability remain uncontrolled. Third, kindergarten-level factors, such as the quality and frequency of physical education programming, may independently shape children’s fitness while also being correlated with family socioeconomic background (e.g., through school-choice patterns in the private/public kindergarten split described in Methods), producing a spurious or inflated association between family-level variables and fitness at the individual level. The present cross-sectional design cannot distinguish the proposed pathway from reverse-causal or common-cause explanations. This uncertainty limits policy and intervention inferences pending evidence from longitudinal or experimental studies.

### Dimensional pattern and cultural context

Among the four dimensions of the HPAE, Behavioral Support (*M* = 3.43) received the highest ratings, while Equipment Availability (*M* = 2.99) received the lowest, as reported in Table 4. This pattern may reflect the Chinese cultural context in which parents often express strong verbal encouragement for their children’s development but may allocate household resources preferentially toward academic enrichment rather than sports equipment (26, 56). The relatively lower equipment scores echo findings from Tandon et al. (25), who reported that the availability of portable play equipment in home-based childcare settings was limited even when outdoor space was adequate. This pattern may also reflect the physical constraints of high-density residential environments characteristic of Chinese urban centers, where compact apartment layouts impose spatial limitations on the storage and use of large-format physical activity equipment such as trampolines, climbing frames, or balance beams.

Notably, all four dimensions showed remarkably similar magnitudes of correlation with the fitness composite (*r* = 0.323 to 0.343, as detailed in Table 1), indicating similar bivariate associations with children’s physical fitness (although, as discussed above, this uniformity is also consistent with the four dimensions largely indexing the same underlying construct). This finding contrasts with studies of older children in Western contexts, where parental logistic support has been identified as the strongest predictor of physical activity (57, 58). The relative uniformity across dimensions may reflect the developmental stage of the participants, as preschool-aged children are more heavily dependent on the immediate home environment and less reliant on organized extracurricular programming. Changes such as providing age-appropriate equipment, designating an indoor play area, or establishing a daily outdoor play routine are possible components for future intervention testing; the present data do not establish that these changes improve children’s physical fitness.

### Practical implications

The findings identify questions for future intervention research. A dual strategy combining support for parental physical activity with changes to the HPAE could be tested, including equipment provision, space arrangement, screen-time rules, and opportunities for shared activity. The present cross-sectional associations, including the near-zero indirect association after adjustment for children’s outdoor activity, organized sport participation, and screen time, do not establish the effectiveness of this strategy. Future trials could compare a combined approach with each component alone and an appropriate control condition, assessing changes in children’s activity and fitness.

Kindergarten-based programs could provide a setting for such testing. Potential components include take-home activity kits, illustrated guides for arranging safe indoor play spaces, and parent workshops on outdoor routines and shared activity. Prior work on family-based interventions and parent–child activity provides a rationale for considering these components (59, 60), but the current study does not determine which components are effective or whether their combination is superior. Feasibility, participation, and changes in objectively measured activity and fitness would require evaluation in prospective intervention studies.

The pandemic period also illustrates the relevance of the home setting. Prolonged home confinement during COVID-19 lockdowns highlighted both the potential and the limitations of the home as a setting for children’s physical activity (61). Studies have demonstrated that children who had access to home-based exercise programs maintained or even improved their physical fitness during school closures (4), a brief illustration of why a supportive home environment can matter for children’s fitness even outside a pandemic context.

### Limitations and future directions

Several limitations should be acknowledged. First, the cross-sectional design precludes causal inferences. The indirect association in the primary model is compatible with a potential pathway but does not establish mediation, and its reduction to approximately zero in the child-activity-adjusted model further limits that interpretation. The temporal ordering of the variables cannot be determined. Longitudinal studies tracking changes in parental physical activity, home environments, and children’s fitness over time are needed to establish temporal precedence. Second, parental physical activity was assessed through self-report using the IPAQ-SF, which is known to overestimate moderate-to-vigorous physical activity relative to objective accelerometer-based measurements (62). The IPAQ-SF also aggregates activity across domains (occupational, transport, household, leisure) rather than distinguishing them, and both parental activity and the home environment were reported by the same responding parent on the same occasion, so common-method variance affecting the a-path (parental activity → home environment) cannot be ruled out; Harman’s single-factor test, which we report, is a relatively weak safeguard against method bias localized to two specific constructs, and the *post hoc* marker-variable adjustment we report relies on an imperfect marker (parental age). This overestimation may have inflated the variance of the parental physical activity variable and potentially attenuated or distorted the magnitude of the observed associations. Future studies should consider incorporating wrist-worn accelerometers to provide more precise and unbiased estimates of parental activity levels, and ideally a marker-variable design to permit direct estimation of common method variance. Third, the HPAE questionnaire, although psychometrically promising in several respects, was developed and validated within the same sample used for hypothesis testing, which risks circularity and optimistic fit statistics (the split-sample cross-validation reported in Methods reduces but does not remove this concern); in addition, the heterotrait-monotrait ratios reported above indicate that its four hypothesized dimensions are not currently well distinguished empirically, so it should be regarded as a preliminary total-score measure that requires cross-validation in independent populations, including test–retest reliability assessment, re-estimation of discriminant validity, and predictive validity evaluation against objectively measured physical activity outcomes. Fourth, the sample was drawn from a single Chinese city, limiting generalizability. Multi-site studies spanning diverse regions and cross-cultural investigations would help clarify whether the observed statistical pathway is culturally specific or universal. Fifth, common method bias, discussed above, is only partially addressed by the Harman’s single-factor test we report; future studies should employ multi-informant designs (e.g., both parents, or teacher report of the child) or objective environmental audits, and only one parent’s activity and perceptions were captured here, leaving the non-responding parent’s activity and potential contribution to the home environment entirely uncaptured despite the plausibly family-level nature of the proposed mechanism. Sixth, although we tested and did not detect moderated mediation by respondent gender or child age group in a supplementary analysis (see Results), these interaction tests are likely underpowered relative to the main-effect tests, so this remains a limitation rather than a settled null result; future investigations should examine whether the statistical pathway operates differently across parent gender and child developmental stage in larger, purpose-designed samples.

Seventh, the supplementary analysis presented above indicates that children’s own outdoor activity time, organized-sport participation, and screen time are strongly associated with all three focal variables and, when added as covariates, remove the statistical evidence for the home-environment mediation pathway (see Results). Because these child-level variables were not part of the *a priori* theoretical model and may themselves be intermediate outcomes rather than confounders, we did not adopt them as covariates in the primary model; however, their uncertain causal position does not justify privileging the primary model. After adjustment for children’s outdoor activity, organized sport participation, and screen time, the indirect association was close to zero and was not statistically significant (a × b = −0.002, 95% CI [−0.013, 0.007]). The data therefore support only a model-dependent pattern consistent with a potential pathway, and future longitudinal or experimental designs are needed to distinguish competing explanations. Other plausible confounders, notably neighborhood walkability and access to parks and play spaces, were not measured, and kindergarten programming quality was accounted for only indirectly, through the kindergarten fixed-effects analysis. The modest explained variance of the primary models (*R*^2^ = 0.055 to 0.142) is consistent with such unmeasured influences.

Eighth, an ICC computed on the age-sex-standardized fitness composite could be close to zero partly by construction, because the standardization procedure itself removes compositional variance between kindergartens. We therefore also examined ICCs on the raw subtest scores. Raw (pre-standardization) subtest-level ICCs were likewise negligible (0.000–0.012 across the six subtests), and a kindergarten-clustered robust standard error re-analysis together with a cluster-level bootstrap (resampling kindergartens) of the primary mediation model produced results consistent with the original single-level analysis (see Statistical analysis and Results). We nonetheless note that with only 23 clusters, cluster-robust inference has known small-sample limitations, and a fully powered multilevel study with a larger number of kindergartens would provide the most definitive test of this question.

Ninth, three of the six fitness subtests (tennis ball catching, continuous jumping in 15 s, and sit-to-walk) represent adapted versions of the classic NPFM-PC items described by Fang and Ho (37), rather than exact replications. The catching subtest is conceptually informed by the MABC-2 framework (38), but the MABC-2 does not directly validate the exact two-handed catching protocol used in the present study. For sit-to-walk, published Timed Up and Go reliability studies (39, 40) used a chair-based starting position, whereas the present study used a floor-seated starting position; this procedural difference means that published TUG reliability data do not directly establish reliability for the floor-based variant, introducing additional measurement uncertainty. The 15-s continuous-jumping variant, to our knowledge, also lacks independently published test–retest reliability data. These adaptations limit direct comparability with studies employing the standard NPFM-PC battery and introduce measurement uncertainty into the fitness composite that may attenuate or inflate the observed associations.

## Conclusion

Among 568 parent–child dyads in urban China, the primary model, adjusted for parental education and family income, showed an indirect association between parental physical activity and preschool children’s physical fitness through the HPAE (a × b = 0.081, 95% CI [0.055, 0.109]). The 48.9% mediated proportion applies only to the primary model. After additional adjustment for children’s outdoor activity, organized sport participation, and screen time, the estimated indirect association was close to zero and was not statistically significant (a × b = −0.002, 95% CI [−0.013, 0.007]). These findings describe an observed pattern of associations consistent with a potential pathway, but do not establish a mediation mechanism or a causal effect of the home environment on fitness. Longitudinal and experimental studies are needed to clarify temporal ordering, determine the role of children’s activity, and test whether supporting parental activity and changing the home environment, separately or together, improves children’s fitness.

## Data Availability

The datasets presented in this article are not readily available because the raw data supporting the conclusions of this article are available from the corresponding author upon reasonable request. Requests to access the datasets should be directed to 13767989570@163.com.
