# Supplementary material for: The home physical activity environment as a potential mediator of the association between parental physical activity and preschoolers’ physical fitness: a cross-sectional study in urban China
Source: Front Public Health. 2026 Oct 28;14:1880097. doi: 10.3389/fpubh.2026.1880097 (PMC13752106; doi:10.3389/fpubh.2026.1880097)
Supplement: Supplementary file 1 [file Table_1.DOCX]

**Table S1. Physical fitness subtest scores by age group and sex**

| **Subgroup** | ***n*** | **Sit-and-reach (cm)** | **Standing LJ (cm)** | **10-m shuttle (s)** | **Ball catch (count)** | **Cont. jump (count/15 s)** | **Sit-to-walk (s)** |
| --- | --- | --- | --- | --- | --- | --- | --- |
| 3-year, Boys | 91 | 11.7 ± 3.0 | 81.4 ± 13.9 | 8.0 ± 0.7 | 4.3 ± 2.0 | 19.3 ± 4.3 | 5.7 ± 0.6 |
| 3-year, Girls | 89 | 12.3 ± 3.2 | 73.9 ± 13.6 | 8.3 ± 0.9 | 4.0 ± 2.1 | 17.8 ± 4.7 | 6.0 ± 0.7 |
| 4-year, Boys | 94 | 11.7 ± 3.2 | 90.7 ± 11.6 | 7.5 ± 0.7 | 5.0 ± 1.7 | 21.6 ± 4.4 | 5.3 ± 0.5 |
| 4-year, Girls | 83 | 11.7 ± 3.3 | 82.3 ± 13.8 | 7.9 ± 0.7 | 4.4 ± 1.8 | 20.1 ± 5.1 | 5.7 ± 0.6 |
| 5-to-6-year, Boys | 118 | 12.4 ± 3.2 | 106.0 ± 14.6 | 6.8 ± 0.7 | 5.9 ± 1.9 | 24.4 ± 4.8 | 5.0 ± 0.6 |
| 5-to-6-year, Girls | 93 | 11.9 ± 3.2 | 95.3 ± 15.2 | 7.4 ± 0.9 | 5.3 ± 1.8 | 22.4 ± 5.0 | 5.4 ± 0.6 |

***Note.*** *Values are M ± SD. LJ = long jump; Cont. = continuous. Shuttle run and sit-to-walk are timed tests (lower = better performance).*

**Table S2. Home Physical Activity Environment Questionnaire: Complete item list**

| **Code / Dimension** | **English Item** | **Chinese Item (中文)** |
| --- | --- | --- |
| **Equipment (5 items, α = .862)** |  |  |
| EQ1 | Our home has ball sports equipment (soccer, basketball, badminton, etc.) | 家中有球类运动器材（足球、篮球、羽毛球等） |
| EQ2 | Our home has riding/gliding equipment (bicycle, scooter, balance bike) | 家中有骑行/滑行类器材（自行车、滑板车、平衡车） |
| EQ3 | Our home has small exercise equipment (jump rope, hula hoop, etc.) | 家中有跳绳、呼啦圈等小型运动器材 |
| EQ4 | Our home has exercise mats suitable for young children | 家中有适合幼儿使用的运动垫/爬行垫 |
| EQ5 | The variety and quantity of sports equipment at home is sufficient | 家中运动器材的种类和数量充足 |
| **Space (4 items, α = .834)** |  |  |
| SP1 | There is enough indoor space at home for the child to run and jump | 家中有足够的室内空间供孩子跑跳活动 |
| SP2 | The community has outdoor spaces suitable for young children | 小区/社区内有适合幼儿活动的户外场所 |
| SP3 | A park or sports field is within walking distance from home | 家附近步行可达公园或运动场 |
| SP4 | Children can safely do outdoor activities around the home | 孩子可以安全地在家周围进行户外活动 |
| **Rules (4 items, α = .802)** |  |  |
| RU1 | We limit our child's daily screen time (TV/phone) | 我们家限制孩子每天看电视/手机的时间 |
| RU2 | We require our child to have outdoor activity time daily | 我们家规定孩子每天必须有一定时间的户外活动 |
| RU3 | We do not allow our child to sit for prolonged periods | 我们不允许孩子长时间连续久坐 |
| RU4 | Our family has rules or agreements that encourage physical activity | 家庭中有鼓励运动的家规或约定 |
| **Support (5 items, α = .878)** |  |  |
| SU1 | I often encourage my child to engage in physical activity | 我经常鼓励孩子参加体育活动 |
| SU2 | I often accompany my child in exercise or outdoor play | 我经常陪伴孩子一起运动或做户外游戏 |
| SU3 | I transport my child to sports classes or training | 我会接送孩子参加体育兴趣班或训练 |
| SU4 | I demonstrate and teach sports skills to my child | 我会示范和教孩子运动技能 |
| SU5 | Family members are positively supportive of the child's sports participation | 家庭成员对孩子参与运动持积极支持态度 |

***Note.*** *All items rated on a 5-point Likert scale (1 = strongly disagree to 5 = strongly agree). α = Cronbach's alpha. PA = physical activity.*

**Table S3. Item-level provenance mapping of the 18-item Home Physical Activity Environment Questionnaire**

| **Code** | **Dimension / Item (English)** | **Primary source** | **Adaptation status** | **Rationale for adaptation or new development** |
| --- | --- | --- | --- | --- |
| EQ1 | Equipment: Ball sports equipment (soccer, basketball, badminton, etc.) | Sirard et al. (2008) | Adapted | Core equipment inventory item retained; badminton added as culturally prevalent in Chinese households |
| EQ2 | Equipment: Riding/gliding equipment (bicycle, scooter, balance bike) | Sirard et al. (2008); Tandon et al. (2012) | Adapted | Balance bike added to reflect contemporary preschool equipment in urban China |
| EQ3 | Equipment: Small exercise equipment (jump rope, hula hoop, etc.) | Sirard et al. (2008) | Adapted | Preschool-appropriate small equipment retained; wording simplified for parent report |
| EQ4 | Equipment: Exercise mats suitable for young children | — | Newly developed (Delphi) | Addresses indoor floor-based play common in high-density Chinese apartments; no direct equivalent in source instruments |
| EQ5 | Equipment: Variety and quantity of sports equipment is sufficient | — | Newly developed (Delphi) | Global sufficiency judgment added to capture overall resource adequacy beyond item-by-item presence |
| SP1 | Space: Enough indoor space at home for the child to run and jump | Tandon et al. (2012) (space items) | Adapted & rewritten | Emphasizes indoor space constraints typical of Chinese urban apartments rather than suburban yards |
| SP2 | Space: Community has outdoor spaces suitable for young children | Hume et al. (2007) | Adapted | Neighborhood outdoor space item reframed to “community/xiaoqu” terminology familiar to Chinese parents |
| SP3 | Space: Park or sports field within walking distance from home | Hume et al. (2007); Tandon et al. (2012) | Adapted | Walking-distance framing retained; “sports field” added for local relevance |
| SP4 | Space: Children can safely do outdoor activities around the home | — | Newly developed (Delphi) | Safety emphasis introduced because traffic and stranger-danger concerns are salient in Chinese urban parenting |
| RU1 | Rules: We limit our child’s daily screen time (TV/phone) | Tandon et al. (2012) | Adapted | Screen-time rule item retained; mobile devices (phones/tablets) added explicitly |
| RU2 | Rules: We require our child to have outdoor activity time daily | Tandon et al. (2012) | Adapted | Strengthened from “encourage” to “require” to reflect the regulatory dimension of family rules |
| RU3 | Rules: We do not allow our child to sit for prolonged periods | — | Newly developed (Delphi) | Explicit sedentary-behavior restriction item written for preschool age group |
| RU4 | Rules: Our family has rules or agreements that encourage physical activity | — | Newly developed (Delphi) | Broader pro-activity family-rule item added to capture general regulatory climate |
| SU1 | Support: I often encourage my child to engage in physical activity | Tandon et al. (2012); Beets et al. (2010) | Adapted | Standard parental encouragement item retained with minor wording localization |
| SU2 | Support: I often accompany my child in exercise or outdoor play | Tandon et al. (2012) | Adapted | Co-participation item retained; potential conceptual overlap with parental PA noted in Methods and tested via robustness check |
| SU3 | Support: I transport my child to sports classes or training | Tandon et al. (2012); Beets et al. (2010) | Adapted | Logistical support item retained; highly relevant given prevalence of preschool enrichment classes in urban China |
| SU4 | Support: I demonstrate and teach sports skills to my child | — | Newly developed (Delphi) | Active skill-modeling item added to capture direct instructional support |
| SU5 | Support: Family members are positively supportive of the child’s sports participation | — | Newly developed (Delphi) | Multigenerational household support item written to reflect co-resident grandparents common in Chinese families |

***Note.*** *Primary source instruments: (1) Sirard et al. (2008) – Home Environment Inventory for Physical Activity and Media Equipment; (2) Tandon et al. (2012) – home environment measures of physical activity, sedentary time, and screen time; (3) Hume et al. (2007) – perceived neighborhood environment (items adapted to home/community scale). “Newly developed” items were generated or substantially rewritten during the two-round Delphi consultation (five experts in early childhood physical education and pediatric public health) and subsequent cognitive interviews with 15 parents of preschool-aged children, specifically to address features of urban Chinese preschool households (high-density apartment living, multigenerational co-residence, and locally salient safety concerns). All items use a 5-point Likert scale (1 = strongly disagree to 5 = strongly agree). α = Cronbach’s alpha in the analytic sample (N = 568). For SU1 and SU3, Beets et al. (2010) is listed as a conceptual reference only: it is a review of parental social support rather than a measurement instrument, and its distinction between motivational support (e.g., encouragement; SU1) and instrumental/logistic support (e.g., transportation; SU3) informed the framing of these two items. The source instrument for both items is Tandon et al. (2012), so both are counted among the 11 items adapted from the three primary source instruments.*

Summary: Of the 18 items, 11 were adapted from previously published instruments (Sirard et al., 2008; Tandon et al., 2012; Hume et al., 2007) and 7 were newly developed through the Delphi and cognitive-interview process to address cultural and contextual features of urban Chinese preschool families. The full Delphi rating sheets and cognitive-interview notes are available from the corresponding author upon request.

**Table S4. Competing confirmatory factor analysis models for the Home Physical Activity Environment Questionnaire (*N* = 568)**

| **Model** | **χ²** | **df** | **χ²/df** | **CFI** | **TLI** | **RMSEA** | **RMSEA 90% CI** | **SRMR** | **Δχ² vs. 4-factor** | **Δdf** | **p** |
| --- | --- | --- | --- | --- | --- | --- | --- | --- | --- | --- | --- |
| Four-factorᵃ | 151.9 | 129 | 1.177 | .996 | .996 | .018 | [.000, .028] | .018 | — | — | — |
| Three-factor (Equipment + Space merged)ᵃ | 161.5 | 132 | 1.224 | .995 | .995 | .020 | [.004, .030] | .018 | 9.68 | 3 | .021 |
| Two-factor (Tangible vs. Social–Regulatory) | 175.0 | 134 | 1.306 | .994 | .993 | .023 | [.012, .032] | .019 | 23.14 | 5 | <.001 |
| Single-factor | 175.4 | 135 | 1.299 | .994 | .993 | .023 | [.012, .032] | .019 | 23.55 | 6 | <.001 |

*Note.* All models were estimated with maximum likelihood in lavaan (Rosseel, 2012) using the same dataset (N = 568) and the same 18 items. Four-factor model: Equipment (5 items), Space (4 items), Rules (4 items), Behavioral Support (5 items). Three-factor model: Equipment and Space merged into a single factor. Two-factor model: tangible resources (Equipment + Space) vs. social–regulatory practices (Rules + Behavioral Support). Single-factor model: all 18 items loading on a single general factor. Δχ² = chi-square difference relative to the four-factor model. ᵃImproper solution: the standardized latent factor correlation matrix was not positive definite. In the four-factor model, three of six latent correlations exceeded 1.0 (Equipment–Rules = 1.026, Space–Rules = 1.012, Rules–Support = 1.002; remaining correlations 0.952–0.994); in the three-factor model, the correlation between the merged Equipment/Space factor and Rules was 1.024. The two-factor (latent r = 0.996) and single-factor models were admissible. Chi-square difference tests nominally favored the four-factor model, but all four models showed close absolute fit with virtually identical practical fit indices (ΔCFI ≤ 0.002), and the four-factor comparison involves an improper solution. Together with the discriminant-validity results reported in the main text (Fornell-Larcker criterion not satisfied; HTMT ratios 0.95–1.02), these results support treating the total score as a broad supportive-environment index rather than as four empirically independent constructs. CFI = comparative fit index; TLI = Tucker-Lewis index; RMSEA = root mean square error of approximation; SRMR = standardized root mean square residual.

**Table S5. Complete regression output for the sensitivity analysis adjusting for child activity variables**

| **Path** | **β** | **B** | **SE(β)** | **SE(B)** | ***t*** | ***p*** | **R²** |
| --- | --- | --- | --- | --- | --- | --- | --- |
| c (total effect) | 0.055 | 0.033 | 0.033 | 0.020 | 1.67 | .095 | .183 |
| a (PA → HPAE) | −0.015 | −0.006 | 0.034 | 0.015 | −0.44 | .661 | .524 |
| b (HPAE → Fitness) | 0.153 | 0.213 | 0.040 | 0.056 | 3.81 | <.001 |  |
| c' (direct effect) | 0.057 | 0.034 | 0.032 | 0.020 | 1.76 | .079 | .203 |
| a × b (indirect) | −0.002 | −0.001 | — | — | — | — |  |
| 95% bootstrap CI |  |  |  |  |  | [−0.013, 0.007] |  |

*Note. N* = 568. Covariates: parental education, family income, child outdoor activity time (min/day), organized sport sessions (per week), and recreational screen time (hours/day). β = partially standardized coefficient (predictor and mediator z-standardized; fitness composite in native age-sex-standardized units). B = fully unstandardized coefficient. Bootstrap = 5,000 resamples. This model corresponds to the "+ Child outdoor, sport, screen" row in Table 4. The indirect effect is not significant, consistent with the interpretation that children's own activity may mediate or confound the PA–HPAE–fitness pathway.

**Table S6. One-way random-effects ANOVA output for ICC computation (K = 23 kindergartens, *N* = 568)**

| **Fitness subtest** | **MSB** | **MSW** | **F** | **df₁** | **df₂** | **ICC** |
| --- | --- | --- | --- | --- | --- | --- |
| Sit-and-reach (cm) | 10.106 | 10.255 | 0.985 | 22 | 545 | 0.000ᵃ |
| Standing long jump (cm) | 364.315 | 307.646 | 1.184 | 22 | 545 | 0.007 |
| 10-m shuttle run (s) | 0.939 | 0.892 | 1.052 | 22 | 545 | 0.002 |
| Tennis ball catching (count) | 4.365 | 3.928 | 1.111 | 22 | 545 | 0.004 |
| Continuous jumping / 15 s (count) | 32.440 | 26.969 | 1.203 | 22 | 545 | 0.008 |
| Sit-to-walk (s) | 0.610 | 0.466 | 1.309 | 22 | 545 | 0.012 |
| Fitness composite (z-score) | 0.563 | 0.538 | 1.045 | 22 | 545 | 0.002 |

*Note.* MSB = between-kindergarten mean square; MSW = within-kindergarten mean square; F = MSB/MSW. ICC(1) = (MSB − MSW) / (MSB + (n₀ − 1) × MSW), where n₀ = N/K = 568/23 = 24.7 (arithmetic mean cluster size). Using instead the unequal-cluster-size adjusted value, n₀ = [N − Σnⱼ²/N] / (K − 1) = 24.64, yields identical ICCs to three decimal places. Kindergarten sizes ranged from 12 to 34. All subtest ICCs were computed on raw (pre-standardization) scores; the composite ICC was computed on the age-sex-standardized z-scores. ᵃ Raw ICC = −0.001; negative values are set to 0.000.
